# COVID-19 Vaccination Acceptance in China after It Becomes Available: A Cross-Sectional Study

**DOI:** 10.3390/vaccines9121398

**Published:** 2021-11-25

**Authors:** Qian Zhou, Tian Tian, Jie Ni, Xiaoheng Zhao, Hong Li, Yili Yang, Yumeng Zhang, Jay Pan

**Affiliations:** 1 HEOA Group, West China School of Public Health and West China Fourth Hospital, Sichuan University, Chengdu 610041, China; zhouqian_97@163.com; 2 Sichuan Health Development Research Center, Chengdu 610041, China; tiantiant77@126.com (T.T.); Jieni82@126.com (J.N.); xiaohengzhao@yeah.net (X.Z.); 3 West China Hospital, Sichuan University, Chengdu 610041, China; lihonghxhx@scu.edu.cn; 4 Institute for Healthy Cities and West China Research Center for Rural Health Development, Sichuan University, Chengdu 610041, China; yyl115@hotmail.com

**Keywords:** COVID-19, vaccine acceptance, SARS-CoV-2, China, health belief model

## Abstract

The outbreak of coronavirus disease 2019 (COVID-19) has led to numerous tragic deaths all over the world. Great efforts have been made by worldwide nations for COVID-19 targeted vaccine development since the disease outbreak. In January 2021, the Chinese government started to provide free vaccination among nationwide communities, which was optional for citizens. As no evidence has been provided so far regarding COVID-19 vaccination acceptance since the initiation of nationwide vaccination, this study aims to investigate COVID-19 vaccination acceptance among Chinese citizens as well as its associated factors as an attempt to bridge such gap embedded in the current literature. An anonymous cross-sectional study was conducted online in March and April 2021 among adults, with the survey questionnaire designed based on the framework of the health belief model (HBM). Information on socio-demographics, risk perception, past pandemic-related experience, awareness of vaccination as well as acceptance of COVID-19 vaccination were collected. Chi-squared test and multi-level regression were performed to distinguish the acceptance between different groups as well as to identify the significant predictors. A total of 3940 participants completed the survey, with 90.6% of the participants reporting strong willingness to get vaccinated. A list of factors were found to be significantly associated with individuals’ acceptance of vaccination, including the region of residence, ethnicity, annual income, whether or not they had experienced a major pandemic event in the past, risk perception of the COVID-19 as well as the awareness of receiving vaccination. Safety concerns about the vaccine (27.7%), concerns about receiving vaccination immediately after newly developed vaccines were released into the market (22.4%) as well as concerns about the potential side effects induced by vaccination (22.1%) were identified as the primary reasons of residents’ resistance against vaccination. Overall, residents demonstrated strong willingness to receive vaccination against COVID-19 in China. However, the improvement of vaccination-related knowledge among Chinese residents should be highlighted as a critical strategy to facilitate the penetration of nationwide vaccination in order to ultimately achieve the establishment of herd immunity in China.

## 1. Introduction

The outbreak of coronavirus disease 2019 (COVID-19) has led to numerous tragic deaths all over the worldwide. It is reported that as of 11 August 2021, confirmed worldwide COVID-19 cases reached above 203 million cases, with a daunting number of deaths, as high as 4.3 million, caused by the pandemic. For this reason, the pandemic has posed a tremendous disease burden on the global population [1,2], and no effective therapy has been developed so far against COVID-19 [3,4]. As a consequence, mask wearing, hand washing, social distancing as well as vaccination have been highlighted as key strategies in response to the worldwide pandemic [5,6,7,8], among which vaccination has been proven to be the most cost-effective means of preventing and controlling infectious diseases [9,10,11]. Given such critical role of vaccination to curb the widespread pandemic, great efforts have been made by worldwide nations since the disease outbreak with the aim of developing safe and effective vaccines against COVID-19 as well as its variants [12,13,14]. As of February 2021, approximately 70 types of vaccines have been tested in clinical trials, with another 20 types of vaccines being tested in phase III clinical trials [15]. In the early 2021, the Chinese government made an announcement on providing free vaccines to its nationwide citizens, while such vaccination program intended for all Chinese residents was not officially required as mandatory. Despite remarkable achievements thus far via the implementation of worldwide vaccination programs intended for curbing the COVID-19 pandemic, issues and challenges persist in this regard. As reported by an earlier study, vaccine hesitancy among worldwide residents has been increasing in the past decade [16], which therefore was proposed by the World Health Organization (WHO) as one of the top 10 global health threats in 2019 [16,17]. Vaccine hesitancy is a complex issue, which could induce vaccine refusal or delay, thus further leading to significantly decreased vaccine coverage rate among residents [18]. Under the context of the COVID-19 pandemic, which demonstrates to be a highly transmissible and fatal disease, it is not difficult to imagine that severe consequence would be induced by residents’ hesitant behaviors towards vaccination. Since the outbreak of COVID-19, multiple studies have been conducted in worldwide nations regarding residents’ acceptance of vaccination, such as a survey conducted in China in March of 2020 with the goal of investigating nationwide residents’ attitudes towards vaccination. Likewise, several similar studies were conducted in France, Malaysia, the UK and other countries in 2020 [4,19,20,21]. These studies have shown that vaccine hesitancy is a common global phenomenon. It is noteworthy that based on several recently published studies, vaccine hesitancy was found to be highly associated with multiple factors, such as age, gender, education level, ethnicity and individuals’ risk perception of the disease [4,20,22,23]. However, most of the previous studies regarding this issue were conducted in the year 2020, when the provision of vaccines as well as the related costs remained unknown in the worldwide range. Moreover, no empirical study has been conducted thus far in terms of exploring residents’ acceptance of vaccination after the vaccine became available, especially under China’s context where free vaccination has become available for nationwide residents since early 2021 without any mandatory obligation posed on residents regarding the acceptance of COVID-19 vaccination. Under such context, an in-depth investigation is urgently needed to understand whether or not residents’ negative attitudes towards vaccination have posed huge obstacles in the actual implementation of the COVID-19 vaccination program in China. In an attempt to bridge such gap embedded in the current literature, this study aimed to understand residents’ willingness to receive the COVID-19 vaccination in China as well as factors associated with vaccination refusal or hesitancy. In order to achieve this goal, an anonymous questionnaire survey was conducted among adult residents from January to March 2021, which was expected to provide evidence-based implications to inform vaccination-related policy-making procedures at the governmental level in order to facilitate the implementation of worldwide vaccination-related interventions against the COVID-19 pandemic in a more effective manner.

## 2. Materials and Methods

### 2.1. Study Sample and Data Collection

An anonymous cross-sectional online survey was conducted in China between 1 March 2021 and 10 April 2021. A stratified sample based on the population distribution and regional economic development was adopted in order to make the sample reflective enough of the nationwide situation in terms of both socio-demographic characteristics and the geographical distribution of populations. Residents aged from 18 to 65 were included for analysis.

### 2.2. Measures

**Figure 1:**
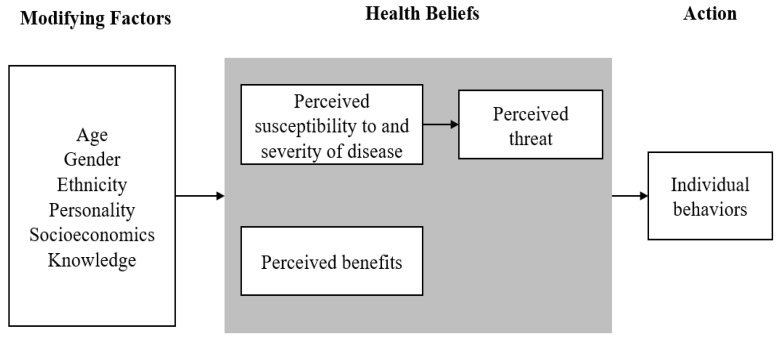
HBM-based model of COVID-19 vaccination acceptance. Notes: this model is adjusted by Rosenstock’s HBM.

The questionnaire was developed based on previous studies and group discussions. After two rounds of expert consultation and pre-survey, the final version of the questionnaire was produced. The questionnaires were delivered and completed by participants via widely adopted online social media platforms, such as WeChat (https://weixin.qq.com/ accessed 11 April 2021) and QQ (https://im.qq.com/index accessed 11 April 2021). Both convenience and snowball sampling strategies were used. The design of the questionnaire followed the framework of the health belief model (HBM), which is one of the most widely adopted theories to investigate individuals’ health behaviors [24]. The theory has also been used in several recently published studies on COVID-19, such as exploring preventive health behaviors among Egyptians [25]. With the key component of the HBM being health beliefs, other modifying factors might also affect individuals’ health beliefs such as sociodemographic factors and health literacy, which further determine individuals’ health behaviors [26]. The model, illustrated in Figure 1, is based on Rosenstock’s HBM, which describes the conceptual framework of COVID-19 vaccination acceptance (Figure 1).

According to the HBM, the survey consisted of three dimensions, namely socio-demographic characteristics, health beliefs and acceptance towards COVID-19 vaccination. Socio-demographic characteristics include age, gender, region of residence, marital status, ethnic, education level, family members, occupation, income and past experience of suffering major infectious disease. Health beliefs referred to individuals’ risk perception of COVID-19 as well as their cognition of COVID-19 vaccine. Risk perception was measured based on the classical risk perception theory proposed by Paul Slovic [27], which classifies risk into four dimensions, namely uncontrollability, severity, unknown and uncertainty. In order to measure the perception risk, six questions were asked, with each aspect measured via a 5-point Likert scale containing “strongly agree”, “relatively agree”, “general”, “not quite agree” and “completely disagree”, with a total score of 30. Following this step, the perception risk was divided into three levels according to the score, including low (6–10), middle (11–20) and high (21–30). Moreover, participants’ cognition of the COVID-19 vaccine was examined by this particular question “Do you think vaccination is important to fight against COVID-19”. Options provided for answers included “very important”, “important”, “general”, “not quite important” and “not important”. When it comes to the acceptance for COVID-19 vaccination, the reason for resistance was also investigated.

Quality control was carried out throughout the procedure. At the design stage, the questionnaire was carefully discussed by the research group. All investigators received professional training before the actual implementation of the research project, while at the data collation stage, a double-blind method was used by two research team members to check the logic and missing items.

### 2.3. Statistical Analysis

Questionnaires completed by participants were extracted from the survey platform, which were further exported into Microsoft Excel 2016 spreadsheets for data cleaning and coding, and subsequently transferred to stata15.0 (StataCorp LLC, 4905 Lakeway Drive College Station, TX USA) for analysis. Health beliefs were summarized as mean and standard deviation (SD). The participants were divided into two groups based on their willingness of receiving vaccination, for which a chi-squared test was performed to identify the differences between groups. Multi-level regression analysis was performed to explore the association between each factor and individuals’ acceptance of COVID-19 vaccine. Individual level variables included demographic characteristics and the health beliefs. The regional level variables included population density, per capital GDP, urbanization rate and the number of COVID-19 cases identified. All independent variables were included in the regression analysis. In the regression outcomes, the odds ratio (OR), standard error (SE) and the 95% confidence interval (CI) were calculated. Two-side *p*-values less than 0.05 were considered as statistically significant.

## 3. Results

### 3.1. Sample Characteristics

Overall, a total of 4067 residents participated in the study, 3940 of whom completed the survey (96.9% effective rate). Among those who completed the questionnaire, 3386 (85.9%) lived in urban areas, more than half of the participants (n = 2293; 58.2%) were male, 87.6% were between 18 and 40 years old and 3.3% (n = 129) were over 50 years old. In addition, 83.1% were married, while about 10% of the participants were single. This study also investigated a couple of minority groups (n = 286, 7.3%), including Tibetan, Yi and Qiang ethnic groups. Most of the participants (n = 3087; 78.4%) had 3 to 5 family members. Regarding educational background, nearly half (n = 2116; 53.7%) of the participants engaged in the survey had obtained a diploma or undergraduate degree. In terms of occupation, people engaged in freelance work accounted for the vast majority of all participants, namely 38.8% (n = 1528). The majority of the respondents (n = 2750; 69.8%) had an annual income ranging from CNY 24,000 to CNY 120,000. Furthermore, 65.7% (n = 2590) of the participants reported having experienced major pandemic events prior to the survey.

### 3.2. Health Beliefs of COVID-19

As previously mentioned, health beliefs consisted of individuals’ risk perception of COVID-19 as well as their cognition of receiving COVID-19 vaccination. The results showed that the overall risk perception of COVID-19 was 17.26 ± 5.59; this reflected a moderate level of risk perception. While about a quarter of respondents (n = 991) perceived high risk of COVID-19 (scored more than 20 points), most of the respondents (61.4%) reported the awareness of average risk (scored between 11–20 points), and another 13.5% (n = 532) perceived the risk level regarding the COVID-19 pandemic as low (scored less than 10 points). Furthermore, the findings indicated that individuals had a cognition score of 3.98 ± 1.13 regarding the necessity of receiving the COVID-19 vaccination, thus suggesting that vaccination was widely accepted by participants as a critical measure against COVID-19. Table 1 shows the mean scores and standard deviations of risk perception of COVID-19 pandemic evaluated in multiple aspects reflective of major negative impacts posed by the pandemic. Specifically, the level of risk perception demonstrated slight variation across different dimensions, with higher levels of risk perceived by individuals in terms of the negative impacts posed by the pandemic on their lives. In terms of the uncontrollability of the pandemic, which mainly referred to whether or not a series of potential risks induced by the pandemic could be mitigated down the road, participants’ responses suggested their perception of relatively lower level risks in this regard, thus indicating participants’ overall satisfaction with the governmental performance in disease control as well as their optimistic attitude towards tackling pandemic-related issues down the road. In attempt to investigate participants’ perception of unknown risk due to the lack of knowledge about the disease, respondents were asked to select an option most reflective of their attitude towards the fact that “scientists do not yet fully understand the COVID-19”, with the score turning out to be 2.79 ± 1.24. In contrast with uncontrollable risks, a relatively higher level of risk was perceived in the uncertainty about the disease, indicating that a major concern about the pandemic resides in that when this life-threatening disease would be eventually eradicated from worldwide communities.

### 3.3. Acceptance of COVID-19 Vaccine

Table 2 presents the frequency distribution and chi-square analysis of COVID-19 vaccination acceptance. Among the total 3940 respondents, 90.6% (n = 3568) were willing to receive COVID-19 vaccination as highly recommended by the Chinese government. As indicated by the statistical outcomes, a list of demographic attributes were found to be significantly associated with individuals’ awareness of accepting COVID-19 vaccination, including age groups, marital status, ethnicity, education level, number of family members, annual income and perceived risk level of COVID-19. Specifically, 90.8% of the participants residing in urban regions demonstrated great willingness to be vaccinated, while respondents who were divorced or widowed showed comparatively less willingness for vaccination (79.0%). Most notably, approximately 240 respondents considered vaccination as an essential procedure against COVID-19 while demonstrating poor willingness to receive the vaccination immediately.

### 3.4. Factors Associated with Vaccination Acceptance

A list of factors were found to be significantly associated with participants’ acceptance of vaccination under the context of COVID-19 based on the adoption of a multilevel logistic model. Table 3 represents the multilevel logistic model outcomes, which showed that individuals’ region of residence, ethnicity, annual income, whether or not they had experienced a major pandemic event prior to COVID-19, whether or not they had certain degrees’ risk perception of COVID-19 as well as individuals’ awareness of receiving COVID-19 vaccination as a critical procedure against the pandemic would significantly affect respondents’ acceptance of vaccination.

After controlling for county-level characteristics, those living in urban areas were found to be more likely to get vaccinated compared with those living in rural areas (OR = 1.59, 95% CI: 1.12–2.24). Past experience of suffering a major pandemic event prior to COVID-19 would significantly increase individuals’ willingness to receive the vaccination (OR = 2.37, 95% CI: 1.77–3.17). In addition, participants tended to be less likely to get vaccinated with decreased risk perception level towards COVID-19 pandemic (OR = 0.32, 95% CI: 0.18–0.58), while those who considered vaccination as an essential procedure against COVID-19 were more likely to be vaccinated (OR = 3.76, 95% CI: 2.76.-5.11). Other detailed results can be found in Table 3.

### 3.5. Specific Reasons for Rejecting Vaccine

**Figure 2:**
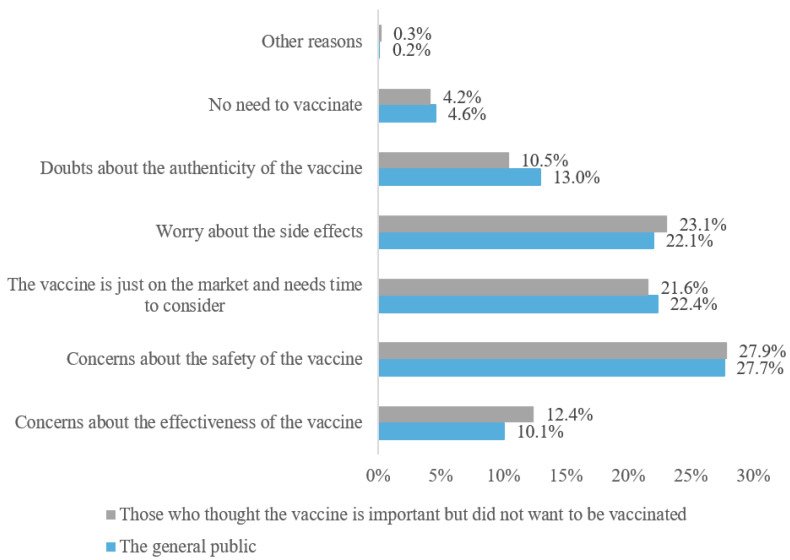
Reasons for rejecting the vaccine.

Among all the 3940 respondents who completed the survey, only about 10% (n = 372, 9.4%) expressed resistance against vaccination. Further factors associated with such resistance were explored, which suggested that respondents were mainly concerned about the safety of the vaccine (27.7%) as well as its side effects (22.1%). Moreover, a number of respondents (22.4%) expressed reluctance to receive the vaccination immediately after vaccines were released into the market as they reported the safety and effectiveness of vaccines needed to be tested over a period of time, while another 10% of respondents were mainly concerned about the actual effectiveness of the vaccine. It can be seen from the figure that only a very small percent (4.2%) of participants considered vaccination as unnecessary in the current situation. In terms of the 240 respondents who considered vaccination as an essential procedure while demonstrating poor willingness to get vaccinated, safety concerns (27.9%) as well as concerns about the side effects potentially induced by vaccination (23.1%) were found to be the primary reasons associated with their resistance against the vaccination (Figure 2). The reasons for rejecting vaccine for these two groups were the same (*p* = 0.93).

## 4. Discussion

Vaccination has been addressed as one of the most cost-effective ways to protect people from infectious diseases, while it is widely accepted that vaccine should be adopted as an indispensable tool for disease control under the context of COVID-19 [9,28,29,30,31]. In January 2021, the Chinese government announced the provision of free vaccination among nationwide communities, which was optional for Chinese citizens. Under such context, this study aimed to investigate Chinese citizens’ acceptance of vaccination as well as its associated factors. To our knowledge, this is the first study to investigate the acceptance of COVID-19 vaccination since free vaccines became available in China [2,4,32,33]. Since the outbreak of COVID-19, the development of effective vaccines against the pandemic has become a competition among worldwide nations, making free vaccination available in multiple countries by the end of 2020 [34]. Striving to accelerate the penetration of COVID-19 vaccination in order to achieve the establishment of herd immunity among populations, a number of countries have implemented a series of strategies for vaccination-related propaganda and education purposes among residents. In China, such strategies included setting up temporary vaccination sites among densely populated regions, rolling out mobile vaccination vehicles or even providing door-to-door vaccination services to those living in rural regions with poor access to medical services [35]. However, the actual effectiveness of all those strategies remains to be validated. In addition, as the number of newly identified cases in China have been sharply reduced via the adoption of multiple measures for disease prevention and control [36], residents in China might hesitate about the necessity of receiving vaccination in this kind of situation.

Based on our findings, 90.6% of the participants engaged in our study demonstrated willingness to get vaccinated. Such outcomes were similar to the findings of a global survey published in June 2020, which reported that the vaccination acceptance rate among Chinese residents was 88.6% [2]. Such findings were also consistent with an anonymous survey in March 2020, which showed that 91.3% of the participants would accept COVID-19 vaccination once vaccines become available [4]. Compared with other countries, the vaccination acceptance rate among Chinese residents was demonstrated to be comparatively higher. For example, a survey conducted in America reported that the highest vaccination rate reached 62% when the vaccine had 80% to 90% effectiveness [37], while another study conducted in Japan reported that about 62.1% of the adults expressed willingness to receive the COVID-19 vaccination [15]. In Europe, such vaccination acceptance ranged from 60% to 80% as indicated by previous studies [38,39,40,41,42,43]. While a study in the Democratic Republic of Congo (DRC) reported that 55.9% of the respondents were willing to get vaccinated [44], another study from Spanish conducted from 10 September to 23 November 2020 reported that 77.56% of the participants engaged in the survey were in favor of receiving vaccination [45]. As a matter of fact, with the constantly expanded range of vaccination coverage from high-risk population groups such as health care workers, to the general public aged from 18 to 60, followed by teenagers and those aged above 60, the number of residents who managed to receive vaccinations in China dramatically increased. As of this writing, over 1.7 billion doses of vaccines have been provided to nationwide residents in China.

It is well known that the spread of infectious diseases largely depends on people’s behaviors, which are often associated with individuals’ health beliefs [46]. In this study, a multi-dimension assessment was conducted to evaluate participants’ health beliefs regarding the COVID-19 pandemic, based on the level of pandemic-related risk perception, which was found to be moderate among Chinese residents with generally satisfying cognition of COVID-19 vaccines. Based on information collected from the survey, participants believed that the COVID-19 was controllable and that scientists have gained a good knowledge of the infectious disease. Nevertheless, the COVID-19 pandemic seemed to have posed significant impact on residents’ life, as the score gained in this aspect was presented to be higher than all the other dimensions proposed in the questionnaire for risk perception assessment. Furthermore, the majority of participants engaged in the survey considered COVID-19 as a fatal disease associated with high risk of death after infection, while uncertainty about the pandemic was generally reported by participants in terms of how long this pandemic would persist. In conclusion, our survey indicated that Chinese residents generally demonstrated optimistic attitudes towards the control of the COVID-19 pandemic across China, while uncertainty about the duration of the pandemic remains a major concern. This can be explained by two reasons. On the one hand, the remarkable achievements made so far in curbing COVID-19 across China can be attributed to a series of disease prevention and control tactics strictly implemented at the governmental level, such as rules and regulations established for transmission control, the provision of policy support as well as support for pandemic-related scientific research [47,48,49,50]. On the other hand, as the widespread disease along with its variants is still creating new cases in the worldwide range, at which point this worldwide pandemic will be eradicated remains unknown [51]. In terms of the awareness of receiving vaccination, most of the participants considered vaccination as a vital approach against COVID-19 for disease prevention and control. Given the pivotal role of vaccination as the key to protecting the worldwide population from COVID-19, it is critical to identify factors associated with the acceptance of vaccination. Based on our survey, multiple determinants of vaccination acceptance were identified, including individuals’ demographic characteristics, past experience of suffering major pandemic events as well as individuals’ cognition of vaccination. Specifically, vaccination against the widespread pandemic tended to be more acceptable for urban residents than rural residents, which might have been influenced by poorer access to medical services in rural regions. In addition, the ethnic minority groups expressed a lower acceptance level towards vaccination than the non-minority group in China, which might be associated with particular ethnic beliefs or local customs, thus indicating the necessity of implementing vaccination programs tailored for the specific needs of ethnic minorities. In addition to individuals’ past experiences of suffering major pandemic events as a contributor to vaccination acceptance level, residents’ risk perception of the pandemic was also found to be a significant contributor to receiving vaccination. Such findings are consistent with previous findings from an Italian study, which showed that individuals with medium or low risk perception levels tended to be more hesitant about receiving the COVID-19 vaccination compared with those with higher levels of risk perception [39,52]. In terms of individuals’ cognition of vaccination, which is believed to be directly associated with vaccination behaviors, it was validated by our findings that participants who considered vaccination as an essential procedure against COVID-19 demonstrated stronger willingness to get vaccinated. These findings serve as potent evidences to support the previously established health beliefs model. Regarding reasons for residents’ resistance against vaccination, safety concerns about vaccines was found to be the primary reason, which was consistent with previous studies, such as a study in Hong Kong, which reported that people had doubts or concerns over the safety of the vaccine [16,53,54,55,56]. Moreover, a number of participants had concerns about the side effects potentially induced by vaccination and believed it was not safe to receive vaccinations immediately after the release of newly developed vaccines before the safety and effectiveness of vaccines were tested over time. This explains why a number of residents expressed reluctance to receive vaccination despite recognizing the importance of adopting vaccines during the pandemic. Our findings are consistent with previous studies that residents’ resistance against vaccination was mainly induced by concerns about safety or potential side effects of vaccines [10,20,57]. It is therefore highly suggested that the improvement of vaccination-related knowledge among residents should be addressed as the key to mitigating public concerns over vaccination so as to reduce residents’ resistance against receiving vaccination. To our knowledge, no existing empirical studies have provided evidence on Chinese residents’ acceptance of vaccination since several types of domestically produced vaccines have become available. Furthermore, this is the first study to analyze vaccination-related behaviors via the adoption of the HBM framework, as well as to measure residents’ risk perception of the pandemic via asking participants a couple of meaningful questions based on the classical risk perception theory instead of merely asking them “the chance of getting COVID-19 in the future” as in previous studies [4,32,58]. Compared with other previous studies in this field, our study should be highlighted in that it had been designed in a way that would facilitate the acquisition of more comprehensive knowledge associated with individuals’ health beliefs of the COVID-19 pandemic. Our study also has some limitations, with the primary limitation being the cross-sectional design, which could only describe residents’ willingness for vaccination at the time point when vaccines became available. As a result, we were not able to compare the level of willingness before and after vaccines were released into the market due to the lack of data regarding participants acceptance of vaccination before the governmental announcement of providing free vaccines. Another limitation was that few responses were collected from the aged residents as a vulnerable population group under the impact of COVID-19 due to their limited access to the survey questionnaire posted via online social medical applications. In addition, the type of vaccine may influence the acceptance of vaccination. As this survey did not collect information on vaccine types and vaccine manufactures; we will explore it in a future study.

## 5. Conclusions

Currently, the widespread COVID-19 pandemic remains a critical challenge for global health under such circumstances; vaccination needs to be implemented worldwide range to facilitate the establishment of vaccine-induced herd immunity among global communities [59]. Despite residents’ willingness to get vaccinated was found to be generally strong as indicated by our findings, the ultimate goal of establishing herd immunity across China calls for greater achievements to be made in the nationwide penetration of vaccination. As suggested by our findings, the improvement of vaccine-related knowledge among Chinese residents should be addressed as an essential strategy to facilitate increased vaccination rates among different communities. Our findings are expected to provide insights on the current states of vaccination implementation in China, as well as to provide evidence-based implications to inform policy-making procedures at the governmental level under the context of the COVID-19 pandemic.

## Figures and Tables

**Table 1:** Individual beliefs of COVID-19.

Dimension		Item	$\bar{x}\pm S$
**Risk perception of COVID-19**	Dread risk	There is a high risk of death when infected with COVID-19	2.93 ± 1.28
		COVID-19 has had a big impact on my life	3.04 ± 1.25
	Uncontrollable	COVID-19 will become increasingly dangerous	2.71 ± 1.20
		If contracted COVID-19, it can affect the next generation	2.80 ± 1.22
	Unknown risk	Scientists do not yet fully understand the COVID-19	2.79 ± 1.24
	Uncertainty	There is no telling how long COVID-19 will continue to prevail	3.00 ± 1.25
**Cognition of COVID-19 vaccine**		Vaccination is important to against COVID-19	3.98 ± 1.13

**Table 2:** Frequency distribution and chi-square analysis of COVID-19 vaccine acceptance.

Variables	Willingness to Accept a COVID-19 Vaccine				
	Yes (N = 3568) N (%)	No (N = 372) N (%)	Total N (%)	Chi-Square	*p*-Value
**Region of residence**					
Rural	492 (88.8)	62 (11.2)	554(14.1)	2.31	0.129
Urban	3076 (90.8)	310 (9.2)	3386 (85.9)		
**Sex**					
Male	2072 (90.4)	221 (9.6)	2293 (58.2)	0.25	0.619
Female	1496 (90.8)	151 (9.2)	1647 (41.8)		
**Age**					
18–25	872 (92.4)	72 (7.6)	944 (24.0)	17.48	0.002
26–30	1276 (91.8)	114 (8.2)	1390 (35.3)		
31–40	982 (87.8)	136 (12.2)	1118 (28.4)		
41–50	319 (88.9)	40 (11.1)	359 (9.1)		
>50	119 (92.3)	10 (7.7)	129 (3.2)		
**Marital status**					
Single	415 (91.0)	41 (9.0)	456 (11.6)	34.82	<0.001
Married	2988 (91.2)	287 (8.8)	3275 (83.1)		
Divorced/widowed	165 (79.0)	44 (21.0)	209 (5.3)		
**Ethnicity**					
Non-minority	3321 (90.9)	333 (9.1)	3654 (92.7)	6.35	0.012
Minority	247 (86.4)	39 (13.6)	286 (7.3)		
**Graduate**					
Middle school and below	521 (85.4)	89 (14.6)	610 (15.5)	45.81	<0.001
High school	987 (90.6)	102 (9.4)	1089 (27.6)		
Associate or bachelor	1960 (92.6)	156 (7.4)	2116 (53.7)		
Master and above	100 (80.0)	25 (20.0)	125 (3.2)		
**Number of family members**					
1	47 (87.0)	7 (13.0)	54 (1.4)	16.29	<0.001
2	257 (89.2)	31 (10.8)	288 (7.2)		
3–5	2824 (91.5)	263 (8.5)	3087 (78.4)		
≥6	440 (86.1)	71 (13.9)	511 (13.0)		
**Occupation**					
Government employee	241 (87.0)	36 (13.0)	277 (7.0)	10.29	0.113
Non-government employee	990 (90.7)	102 (9.3)	1092 (27.7)		
Flexible-job worker	1402 (91.7)	126 (8.3)	1528 (38.8)		
Self-employed	503 (88.6)	65 (11.4)	568 (14.4)		
Farmer	87 (88.8)	11 (11.2)	98 (2.5)		
Student	271 (91.9)	24 (8.1)	295 (7.5)		
Retired/unemployed	74 (90.2)	8 (9.8)	82 (2.1)		
**Annual income**					
CNY24,000	417 (92.1)	36 (7.9)	453 (11.5)	70.41	<0.001
CNY24,000–60,000	1406 (93.7)	95 (6.3)	1501 (38.1)		
CNY60,000–120,000	1127 (90.2)	122 (9.8)	1249 (31.7)		
CNY120,000–240,000	441 (86.1)	71 (13.9)	512 (13.0)		
CNY240,000–360,000	134 (80.7)	32 (19.3)	166 (4.2)		
Above CNY360,000	43 (72.9)	16 (27.1)	59 (1.5)		
**Experienced major infectious events**					
No	1206 (89.3)	144 (10.7)	1350 (34.3)	3.60	0.058
Yes	2362 (91.2)	228 (8.8)	2590 (65.7)		
**Perceived risk of COVID-19**					
High (21–30)	514 (96.6)	18 (3.4)	532 (13.5)	34.50	<0.001
Middle (11–20)	2188 (90.5)	229 (9.5)	2417 (61.4)		
Low (6–10)	866 (87.4)	125 (12.6)	991 (25.1)		
**Do you think vaccination is important to against COVID-19**					
Not important	330 (71.4)	132 (28.6)	462 (11.7)	224.01	<0.001
important	3238 (93.1)	240 (6.9)	3478 (88.3)		

Notes: 1 CNY = 0.16 USD.

**Table 3:** Multi-level analysis of factors influencing vaccination acceptance.

Characteristics	OR	SE	*p*-Value	95%CI
**Region of residence**				
Rural	Ref			
Urban	1.59	0.28	0.009	1.12–2.24
**Sex**				
Male	Ref			
Female	1.19	0.15	0.156	0.93–1.53
**Age**				
18–25	Ref			
26–30	1.13	0.24	0.566	0.75–1.71
31–40	0.86	0.19	0.475	0.56–1.31
41–50	0.72	0.20	0.229	0.43–1.23
>50	1.15	0.51	0.750	0.49–2.73
**Marital status**				
Single	Ref			
Married	1.28	0.35	0.372	0.75–2.19
Divorced/widowed	0.58	0.19	0.103	0.29–1.12
**Ethnicity**				
Non-minority	Ref			
Minority	0.63	0.14	0.038	0.41–0.98
**Graduate**				
Middle school and below	Ref			
High school	1.35	0.24	0.101	0.94–1.92
Associated or bachelor	1.40	0.24	0.050	0.99–1.96
Master and above	0.72	0.23	0.300	0.39–1.34
**Number of family members**				
1	Ref			
2	1.50	0.77	0.428	0.55–4.11
3–5	1.69	0.80	0.268	0.67–4.29
≥6	1.21	0.59	0.693	0.46–3.18
**Occupation**				
Government employee	Ref			
Nongovernment employee	1.06	0.28	0.782	0.66–1.71
Flexible-job worker	1.14	0.27	0.564	0.72–1.85
Self-employed	0.94	0.25	0.802	0.56–1.57
Farmer	1.08	0.47	0.856	0.46–2.54
Student	1.23	0.59	0.666	0.48–3.16
Retired/unemployed	0.68	0.34	0.440	0.25–1.83
**Annual income**				
CNY24,000	Ref			
CNY24,000–60,000	0.77	0.24	0.405	0.41–1.43
CNY60,000–120,000	0.59	0.19	0.105	0.31–1.12
CNY24,000–60,000	0.63	0.21	0.169	0.32–1.21
CNY240,000–36,000	0.59	0.23	0.185	0.28–1.28
Above CNY360,000	0.38	0.18	0.040	0.15–0.96
**Experienced major infectious events**				
No	Ref			
Yes	2.37	0.35	<0.001	1.77–3.17
**Perceived risk of COVID-19**				
High (21–30)	Ref			
Middle (11–20)	0.37	0.11	<0.001	0.17–0.65
Low (6–10)	0.32	0.09	<0.001	0.18–0.58
**Do you think vaccination is important to against COVID-19**				
Not important	Ref			
Important	3.76	0.59	<0.001	2.76–5.11
**Number of confirmed or suspected cases in the county**	0.99	0.03	0.657	0.93–1.05
**Population density**	1.00	0.01	0.940	0.99–1.01
**Urbanization rate**	1.01	0.013	0.481	0.98–1.03

Notes: 1 CNY = 0.16 USD.

## Data Availability

All data in the study are available from the corresponding author by request.
